# Segmentation of Cerebral Small Vessel Diseases-White Matter Hyperintensities Based on a Deep Learning System

**DOI:** 10.3389/fmed.2021.681183

**Published:** 2021-11-25

**Authors:** Wei Shan, Yunyun Duan, Yu Zheng, Zhenzhou Wu, Shang Wei Chan, Qun Wang, Peiyi Gao, Yaou Liu, Kunlun He, Yongjun Wang

**Affiliations:** ^1^Department of Neurology, Beijing Tiantan Hospital, Capital Medical University, Beijing, China; ^2^National Center for Clinical Medicine of Neurological Diseases, Beijing, China; ^3^Beijing Institute for Brain Disorders, Beijing, China; ^4^Laboratory of Translational Medicine, Chinese PLA General Hospital, Beijing, China; ^5^Key Laboratory of Ministry of Industry and Information Technology of Biomedical Engineering and Translational Medicine, Chinese PLA General Hospital, Beijing, China

**Keywords:** masking white matter hyperintensities, deep learning, neural network, segmentation, clinical evaluation

## Abstract

**Objective:** Reliable quantification of white matter hyperintensities (WHMs) resulting from cerebral small vessel diseases (CSVD) is essential for understanding their clinical impact. We aim to develop and clinically validate a deep learning system for automatic segmentation of CSVD-WMH from fluid-attenuated inversion recovery (FLAIR) imaging using large multicenter data.

**Method:** A FLAIR imaging dataset of 1,156 patients diagnosed with CSVD associated WMH (median age, 54 years; 653 males) obtained between September 2018 and September 2019 from Beijing Tiantan Hospital was retrospectively analyzed in this study. Locations of CSVD-WMH on the FLAIR scans were manually marked by two experienced neurologists. Using the manually labeled data of 996 patients (development set), a U-shaped novel 2D convolutional neural network (CNN) architecture was trained for automatic segmentation of CSVD-WMH. The segmentation performance of the network was evaluated with per pixel and lesion level dice scores using an independent internal test set (*n* = 160) and a multi-center external test set (*n* = 90, three medical centers). The clinical suitability of the segmentation results, classified as acceptable, acceptable with minor revision, acceptable with major revision, and not acceptable, was analyzed by three independent neuroradiologists. The inter-neuroradiologists agreement rate was assessed by the Kendall-W test.

**Results:** On the internal and external test sets, the proposed CNN architecture achieved per pixel and lesion level dice scores of 0.72 (external test set), and they were significantly better than the state-of-the-art deep learning architectures proposed for WMH segmentation. In the clinical evaluation, neuroradiologists observed the segmentation results for 95% of the patients were acceptable or acceptable with a minor revision.

**Conclusions:** A deep learning system can be used for automated, objective, and clinically meaningful segmentation of CSVD-WMH with high accuracy.

## Introduction

White matter accounts for approximately half of the adult cerebral hemisphere volume, and it primarily contains myelinated axons that connect various gray matter areas of the cerebral cortex and subcortical regions with each other (1). White matter lesions damage this connectivity, leading to an interruption in communication between different functional areas, which ultimately manifests in a form of various neurobehavioral disorders (1, 2).

White matter hyperintensity (WMH), or leukoaraiosis are characteristic lesions of the white matter that appear as hyperintense regions on the fluid-attenuated inversion recovery (FLAIR) magnetic resonance images (MRI) (3–6). Clinically, WMHs can be caused by many conditions, such as plaque accumulation in the white matter small vessels, small vessel inflammation, toxicity after medication use, genetic white matter diseases, infections, demyelinating diseases, metabolic diseases, tumors, brain trauma, and persistent chronic damage in white matter small vessels (4). Matsue and others considered that these imaging findings correspond to a series of histological changes. For example, histological analysis revealed that the ventricle's high signal corresponded to a pale myelin sheath, perivascular proliferation, a discontinuous inner layer of ependyma, and increased subependymal glia. The hyperintensity in the deep and subcortical white matter has been primarily observed as a result of the pale myelin sheath and perivascular hyperplasia. Perivascular hyperplasia has been mainly found in the frontal and/or apical subcortical white matter (4, 7–13). The diameter of hyperplastic areas was usually <3 mm and had an obvious boundary. The diffused white matter lesions (WMLs) in Binswanger's disease are characterized by a pale myelin sheath and tissue thinning due to the loss of myelin sheaths and axons. All of the above WMLs show different degrees of arteriosclerosis (12, 13).

Although WMLs are closely related to cerebrovascular diseases and vascular risk factors, their pathogenesis remains largely unclear and they can be caused by multiple factors (14). WMHs have been observed to be the main manifestation of cerebral small vessel disease (SVD) and they are important factors in the indication of stroke, dementia, and aging (7–13). Additionally, WMHs have been observed to be prevalent in aged people (15).

At present, the Age-related White Matter Changes (ARWMC), Fazekas, modified Scholten's, and Ylikoski scales are widely used in clinical practice (16–18). Existing quantitative methods are time-consuming, laborious, and subjective. Currently, deep convolutional neural networks (CNNs) have been shown to be useful and effective in medical applications. Thus, a highly accurate system for automatic segmentation of WMH aid neuroradiologists in timely quantitative assessment of WMH and significantly reduce the time required for diagnoses (4, 19–22).

In this work, we propose a deep learning system (DLS) for efficient, objective, and automatic prediction of WMH from the FLAIR images. We compare the proposed DLS with the state-of-the-art deep learning architectures and validate its performance using two independent multi-center test datasets. Finally, to analyze the clinical utility of the proposed DLS and check its acceptance by clinicians, we perform a qualitative analysis whereby three clinical neuroradiologists assess the accuracy and quality of the WMH segmentation on four levels, viz: acceptable, acceptable with minor revision, acceptable with major revision, and not acceptable.

## Materials and Methods

The study was approved by the Ethics Committee of the Beijing Tiantan Hospital in accordance with the Helsinki Declaration. Written informed consent from the participants was not required for participation in this study.

### Study Design and Participants

This study retrospectively analyzed the data from 1,156 patients diagnosed with the CSVD associated WMH admitted to the Beijing Tiantan Hospital between September 2018 and September 2019. The patients with a mention of WMH in their electronic health records (EHRs) were reviewed by clinicians for the presence of WMH and the patients with confirmed WMH were included in this analysis. Patients with poor FLAIR image quality were excluded from the analysis. The included patients were randomly divided into a development dataset (*n* = 996, ~85% of the data) and an independent internal test dataset (*n* = 160). Furthermore, for external validation of the segmentation performance, 90 randomly selected patients with clinically diagnosed WMH from the Third China National Stroke Registry (CNSR-III) study were included in the analysis as an external test dataset.

### Data Distribution

#### MRI Acquisition

All the patients were reviewed for the availability of good quality FLAIR images. The scans were acquired from multiple different scanners with a field strength of either 1.5T or 3T according to the clinically used FLAIR collection protocol. The analyzed images had an axial thickness between 0.55 and 1.2 mm and the sagittal and coronal view spacings were between 0.43 and 0.9 (equal along both the planes).

#### Manual Annotation of the WMH

In total, we included 34,228 T2-FLAIR images from 1,156 patients from Beijing Tiantan Hospital with labeled segmented WMHs. In this data set, we labeled 12,087 small leukoencephalopathies (<20 plex^*^spacing), 14,759 medium leukoencephalopathies (between 20 and 150 plex^*^spacing) and 4,003 large leukoencephalopathies (over 150 plex^*^spacing).

For clinical evaluation data set included 90 patients' T2-FLAIR images from three other hospitals across China, which were included in The Third China National Stroke Registry (CNSR-III). Additional detailed information about the lesion sizes can be found in Table 1. Each volumetric MRI had a vertical spacing between 0.55 and 1.2 mm. For each image, the spacing along the x- and y-directions varied from 0.43^*^0.43 to 0.9^*^0.9 mm^2^ between consecutive pixels. The distribution of pixel spacings for each data set is shown in Table 2.

**Table 1:** Data distribution in the manuscript.

		**Positive number**
DLS development	Traning set	870 patients
	Validation set	126 patients
	Inner test set^#^	160 patients
	Summary	1,156 patients
Clinical evaluation	Test data set	90 patients
	Summary	1,246 patients

# *inner test set used for the code optimizzatio program only*.

*Test data set used for the clinical evaluation only*.

**Table 2:** Validation Test 1.

**Data set**	**Lesion size**	**Percentage** **correctly** **labeled (*n*,%)**	**Dice**
**Data set 1**			
Small	<20 (plex^*^spacing)	462 (64.71%)	
Medium	20 ~ 150 (plex^*^spacing)	(80.09%)	
Large	>150 (plex^*^spacing)	39 (96.12%)	0.722
**Data set 2**			
Small	<20 (plex^*^spacing)	601 (68.37%)	
Medium	20 ~ 150 (plex^*^spacing)	909 (82.86%)	
Large	>150 (plex^*^spacing)	325 (96.73%)	0.776
**Date set 3**			
Small	<20 (plex^*^spacing)	361 (50.14%)	
Medium	20 ~ 150 (plex^*^spacing)	425 (68.77%)	
Large	>150 (plex^*^spacing)	234 (92.49%)	0.722

#### Development of Deep Learning System for WMH Segmentation

**Figure 1:**
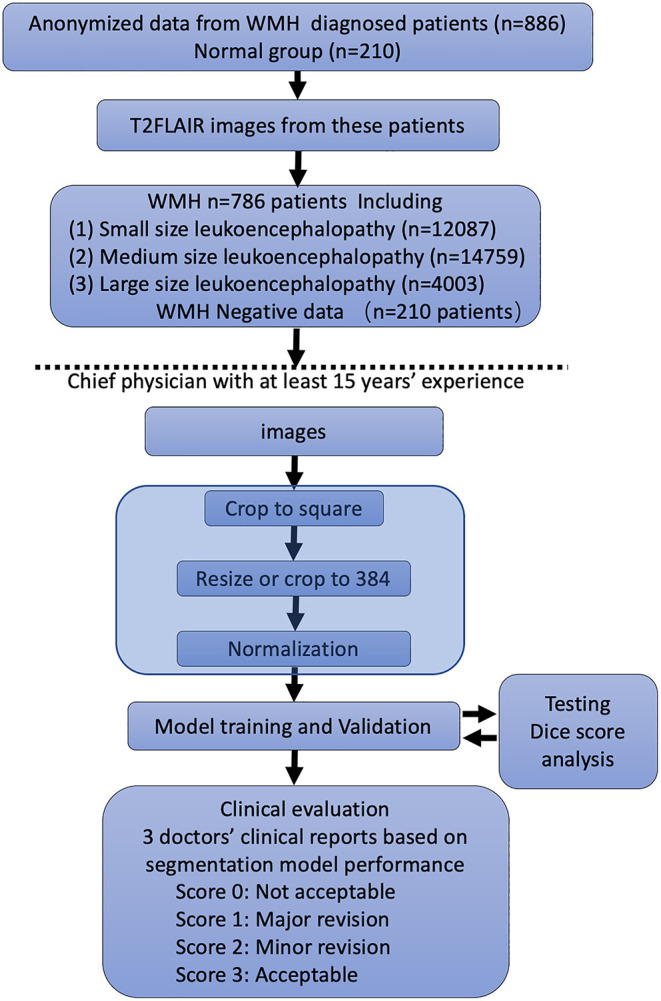
Flowchart of the distribution of patients in the training and clinical evaluation steps. The distribution and classification of all samples in each step was used for the model training and clinical evaluation steps.

For automatic segmentation of the WMH, we developed a deep learning system using the data from the training dataset along with manual annotations (Figure 1). The deep learning system consisted of a four layered modified U-Net architecture which is presented in Figures. The architecture was trained using 996 patients' data from the development dataset. The model was designed to predict a 2D lesion mask using 2D axial slices of FLAIR images. The FLAIR images were first preprocessed by scaling the global (3D) image intensities to follow a standard normal distribution (mean of 0, and standard deviation of 1). Next, images were zero-padded to obtain square-shaped images in the axial plane. The images were next transformed to have uniform axial dimensions of 384 × 384 pixels either using bilinear interpolation (for images with dimensions smaller than 384 × 384 pixels) or using the center crop technique (for images with dimensions larger than 384 × 384 pixels). The decision to center crop the larger images was taken to preserve the spatial resolution of the image which was observed to crucial in the detection of small lesions.

The model was trained using the above preprocessed 2D axial slices of T2 Flair scans (input shape: 384 × 384 × 1) with an Adam optimizer for 200 epochs using a cross-entropy loss and a batch size of 32. The initial learning rate was set to 3 × 10^−4^. To increase the generalizability of the model, data augmentation strategies including vertical flip, horizontal flip, rotation, contrast enhancement, scaling, translation, and addition of Gaussian noise were randomly applied to the images during the training process. The learning rate was modulated based on the dice score on the reserved validation set (*n* = 126 patients from the development dataset). The learning rate was reduced by 10% if the validation set dice score did not improve for 30 consecutive epochs. To avoid model overfitting, the training was stopped if the validation set dice did not improve for 60 consecutive epochs. After the completion of training, the model with the highest dice score on the validation set was selected as a final segmentation model. This model was then used for automatic segmentation of WMH in the external and internal test datasets. The complete 3D WMH mask for each patient was computed by concatenating the 2D WMH masks from all the axial slices.

#### Performance Evaluation of the DLS

The segmentation performance of the proposed DLS was assessed using per-pixel precision, recall, dice score, and accuracy. The precision was defined as the total number of correctly predicted WMH pixels divided by the total number of pixels predicted to be of WMH. The recall was defined as the total number of correctly predicted WMH pixels divided by the total number of WMH pixels in the ground truth segmentation. The dice score was calculated as 2^*^precision^*^recall/(precision + recall). Also, based on the precision and recall, the receiver operating characteristics (ROC) curves were constructed and the area under the ROC was calculated. All these metrics were calculated for each patient and final results on the entire dataset were calculated as the arithmetic mean of the per-patient value. Also, the dice score was independently calculated for small, medium, and large lesions.

Also, the dice score is biased toward the correct prediction of large WMH and by correctly segmenting one large WMH the model can have a high dice score despite it missing multiple small WMH. Therefore, considering the importance of correct segmentation of small WMH, we also employed a lesion-wise precision, recall, dice score as a performance measure. In the lesion-wise analysis, a lesion was said to be correctly identified if at least 40% of the lesioned pixels were correctly marked by the prediction model. In this manner, by counting the correctly identified lesions, and missed lesions, the lesion dice score, precision, and recall were calculated.

Lastly, using the above evaluation metrics, we compared the segmentation performance of the proposed DLS with the state-of-the-art WMH segmentation architectures named U-Resnet and 3D-unet. The architectures were constructed according to the best settings proposed by the respective authors and were trained using the same training data as that of the proposed DLS.

#### Clinical Evaluation of the Proposed DLS

To analyze the clinical utility of the proposed DLS and assess its acceptance by clinicians, we performed a qualitative clinical analysis. In this analysis, three expert neuroradiologists with more than 7 years of experience independently assessed the WMH segmentation results of the proposed DLS for the 90 patients from the external test set. Each neurologist was instructed to rate the segmentation quality of the proposed DLS into four grades, with each of them being defined as:

Grade I (perfectly acceptable, score 4): no missed lesions and <5% mismatch between the predicted and the ground-truth lesions.
Grade II (acceptable with minor revision, score 3): small lesions: 1-4 missed lesions and <10% mismatch for predicted lesions; medium lesions: <2 missed lesions and <5% mismatch; large lesions: no missed lesions.
Grade III (acceptable with major revision, score 2): small lesions: more than four missed lesions and <50% mismatch; medium lesions: more than two missed lesions. Large lesions: more than 30% mismatch.
Grade III (not acceptable, score 1): small lesions: more than eight missed and more than 50% mismatch; medium lesions: more than two missed; Large lesions: more than 30% missed and more than 30% mismatch.

### Statistical Analysis

The inter-radiologist agreement rate and the Kendall W statistic were calculated for each validation using SPSS software (version 20.0). One-way ANOVA with *post hoc* Tukey's test was applied to assess the differences between each group. Statistical significance was considered at *p* < 0.05. ROC curve and AUC score are performed for the segmentation analysis (https://www.kaggle.com/kmader/use-roc-curves-to-evaluate-segmentation-methods).

## Results

### Baseline Imaging Characteristics

The FLAIR images from the 1,156 patients contained a total of 34,228 2D axial slices. In these slices, following manual annotations, a total of 12,087 small, 14,759 medium, and 4,003 large WMH lesions were identified (Figure 1). The distribution of the lesion size was observed to be consistent across the development, internal test, and external test datasets.

### Segmentation Performance of the DSL

**Figure 2:**
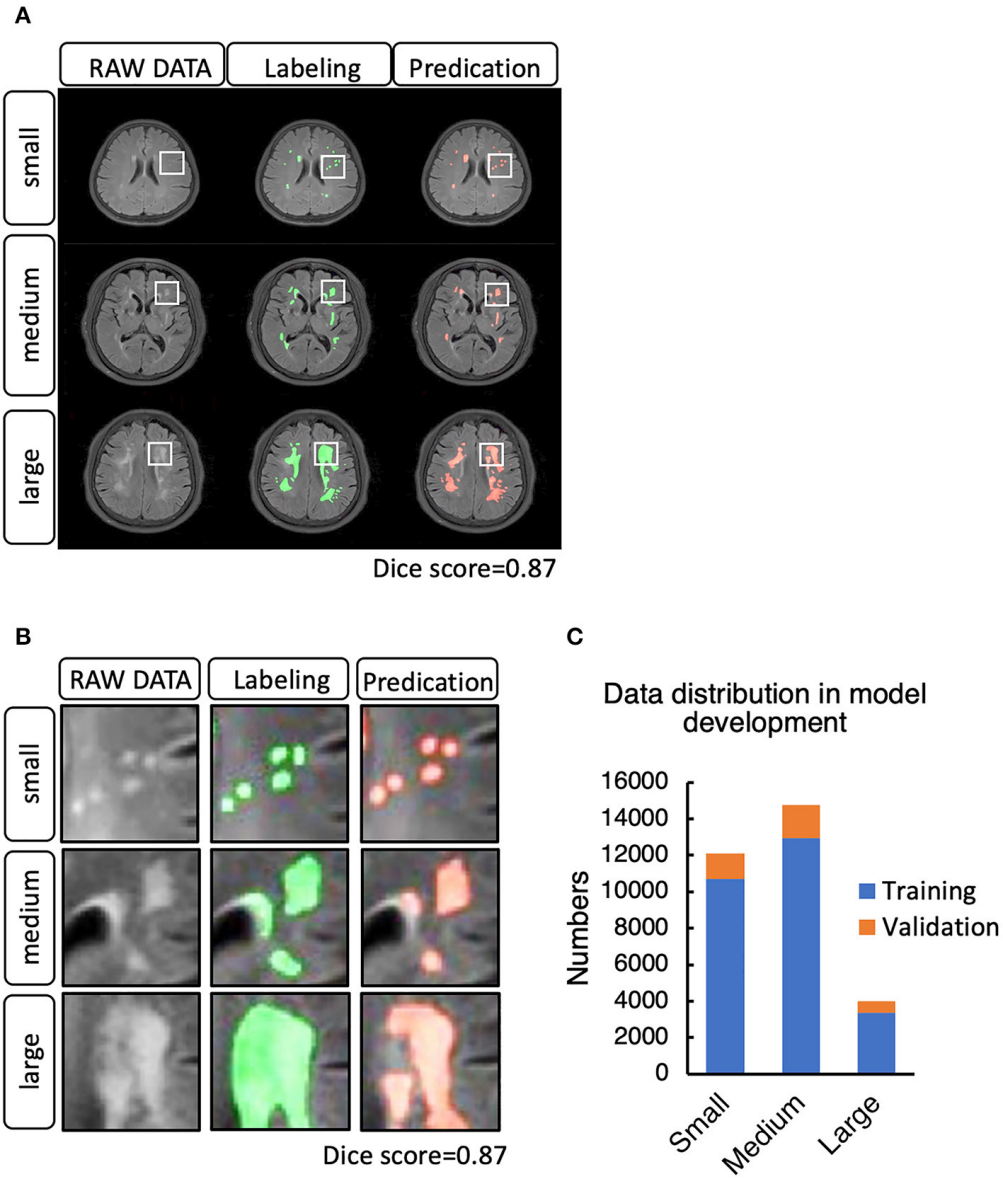
**(A)** Example cases of white matter hyperintensities (WMHs) labeled manually and by the DLS system. **(B)** WMH lesion distribution in the training and validation step. **(C)** Data distribution in the model development for training and validation.

**Figure 3:**
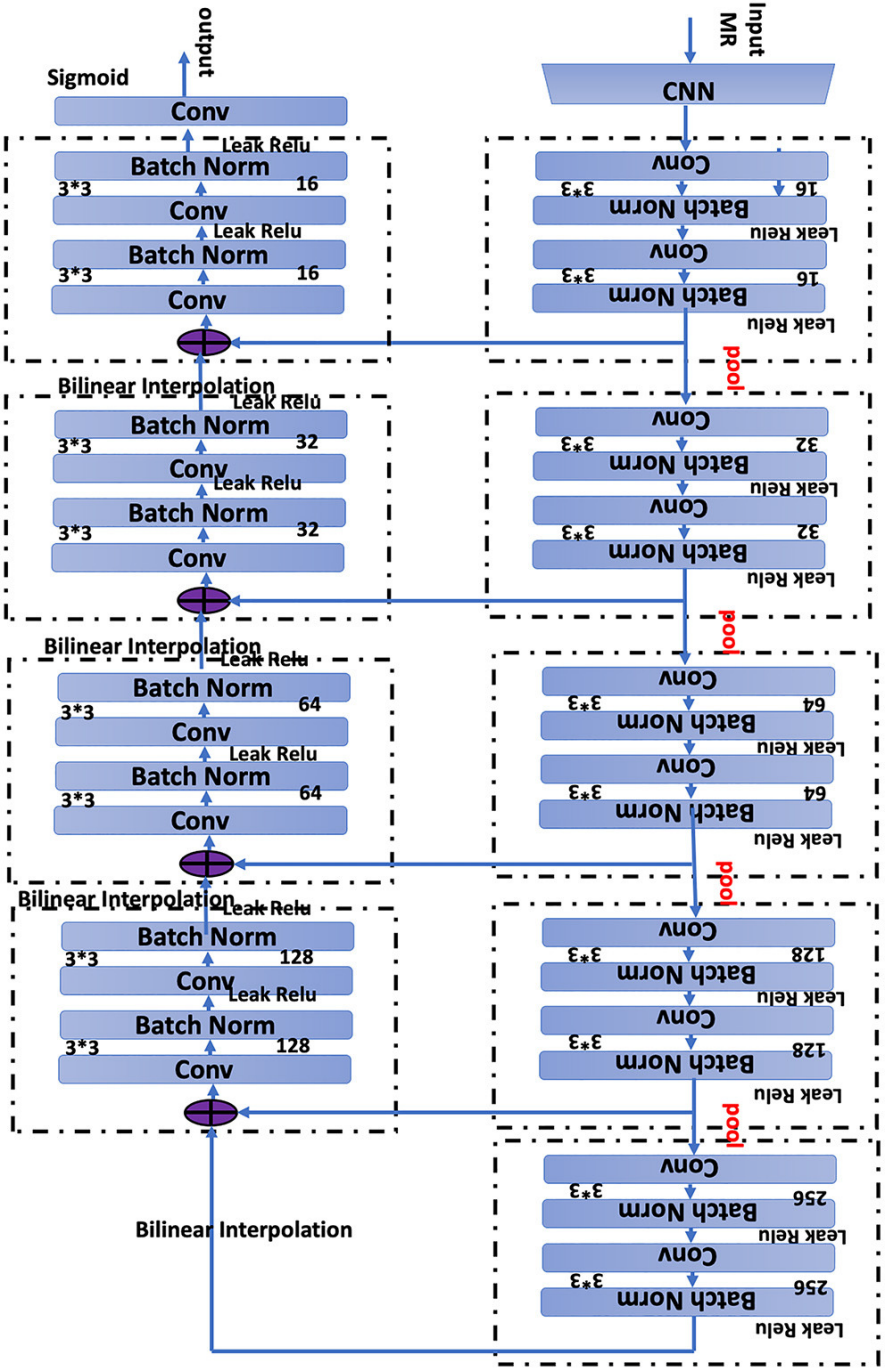
Network architecture of the proposed two-dimensional (2D) convolutional neural network (CNN). The network has 19 layers integrating nine Convolution blocks. Bilinear interpolating arrows indicate up sampling operations to make predictions for the segmentation task. The pool arrow indicates the down sampling operation to gradually increasing the receptive field for the segmentation task. Concatenate connections are used to fuse Multi-scale features in the network. Batch normalization is a linear transformation of the features performed to reduce the covariance shift, thus speeding up the training procedure. Convolution bars indicate the convolution operation, which computes the features. The number 16, 32, 64, 128, 256 indicates the number of channels in that layer, and 3·3·3·3·3·3 denotes the size of the 2D CNN kernels.

**Figure 4:**
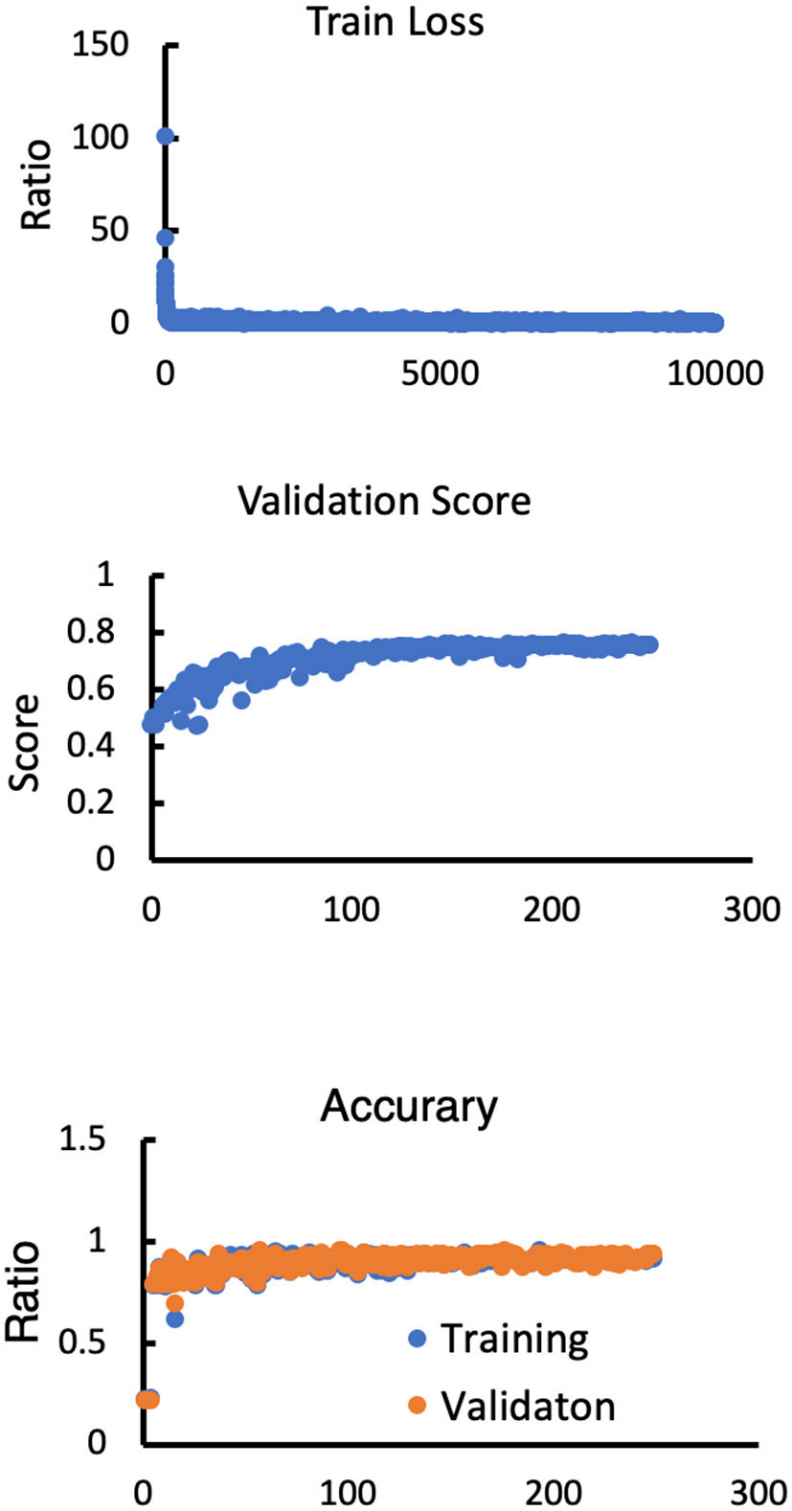
Model performance in terms of the training loss, validation score, training accuracy and validation accuracy.

To set up the DLS, the images were first labeled manually. In summary, we manually labeled ~12,087 small lesions, 14,759 medium lesions, and 4,003 large lesions for training and validation (Figures 2A-C; Table 2). The network architecture of the proposed 2D convolutional neural network is shown in Figure 3. The model quality control parameters could be fond in Figure 4. More detailed information on the network can be found in the Network Architecture portion of the Methods section. After training and validation, the DLS was tested with the testing data set. The accuracy of the DLS-generated masking is represented in Figures 2A,B, with a Dice score of 0.87.

In the segmentation of WMH lesions, the proposed DLS achieved average pixel-wise dice score, precision, and recall of 0.711, 0.789, and 0.647 on the external test set. The lesion wise dice score, precision, recall, and accuracy achieved by the model were 0.735, 0.725, and 0.653 on the external test set. Also, the dice score of the model in segmentation of small, medium, and large WMH was 0.53, 0.82, and 0.96, respectively. Furthermore, in the lesion level analysis on the external test set, the model could correctly identify 61.07, 77.24, and 95.11% of the small, medium, and large lesions, respectively, and the detailed results of this analysis are presented in Table 2. A few examples of WMH segmentation using the proposed system are presented in Figure 2. Also, in the segmentation of the WMH, the proposed DLS achieved a mean AUC of 0.9959 on the external test dataset (Table 3).

**Table 3:** Models head-to head analysis (Data set 2)/Correct labled ratio.

**Models**	**Small lesions** **(879)**	**Medium lesions** **(1,097)**	**Large lesions** **(336)**
U-Resnet	551, 62.68%	863, 78.66%	321, 95.54%
3D-unet	365, 41.52%	778, 70.92%	328, 97.62%
Our model	601, 68.37%	909, 82.86%	325, 96.73%

Lastly, the average pixel-wise dice score achieved by the UresUnet and 3D-unet networks on the external datasets were 0.584, and 0.623, respectively, and these were worse than the performance of the proposed DLS. Beside the preprocess is also import in the DLS development. For more detail information about the models head-to-head analysis in Table 4.

**Table 4:** Models head-to head analysis.

	**Our model**	**Our model** **no preprocess**	**U-Resnet**	**3D-unet**
ACC.	0.97	0.906	0.97	0.93
Sensitivity	0.7244	0.5706	0.6024	0.6499
Specificity	0.9989	0.9998	0.9998	0.9997
AUC	0.9959	0.9944	0.9958	0.9896

All the testing data are summarized in Tables 2, 3, representing the relabeling results between the DLS tool and the experts (percentage correctly labled rato). From the table, we can see that the manual image labeling is precise and perfectly matches the contouring with the true signaling. This is because the labeling tool and pixels could not be well-controlled when manually drawing the labeling. Thus, the Dice score does not perfectly reflect the DLS segmentation result. These data can only support DLS training and validation. Visually, we checked all the data and found a strong concordance between our DLS and human experts for lesion contouring but, as mentioned above, with low Dice scores.

### Clinical Assessment of the DLS Segmentation

**Figure 5:**
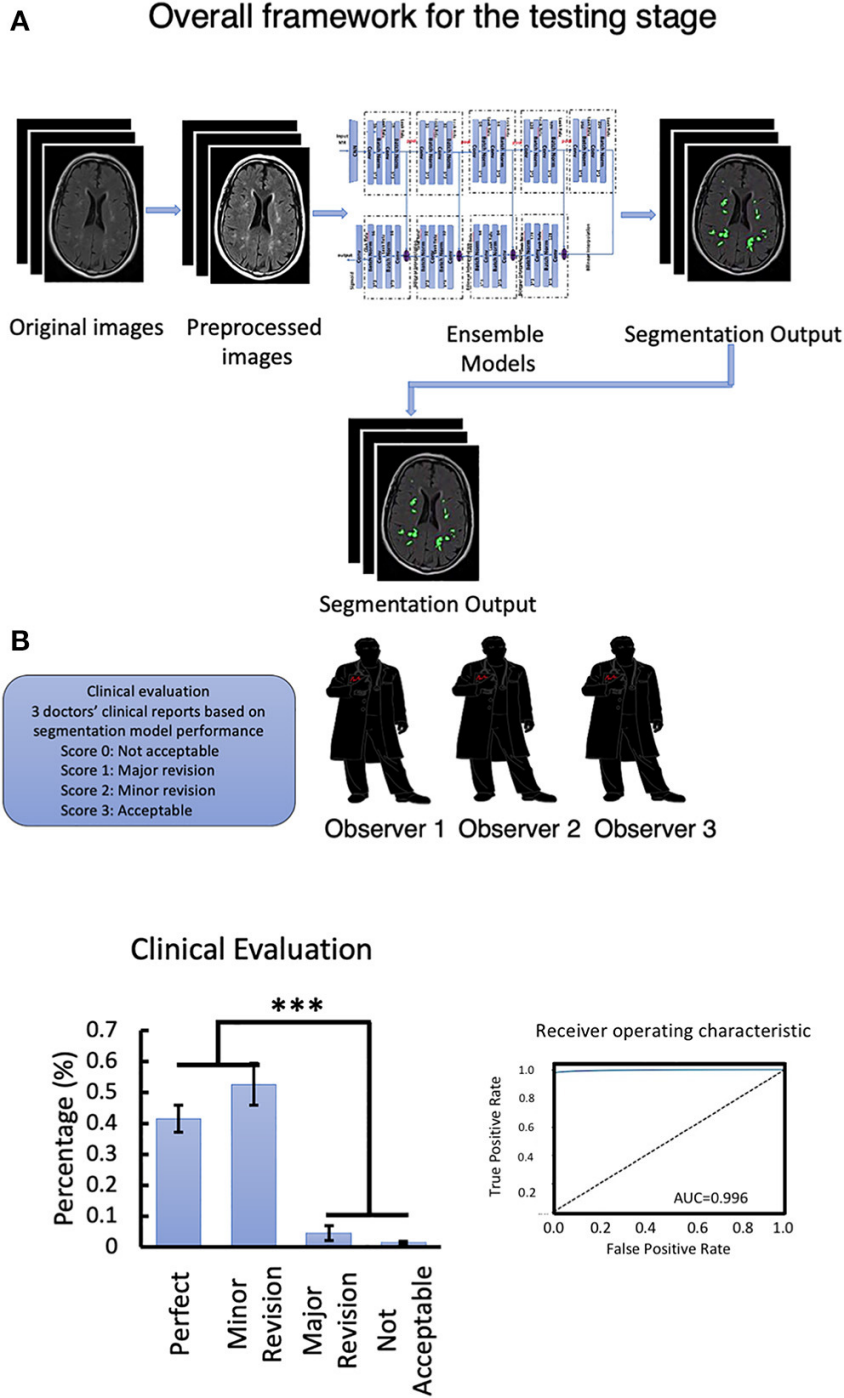
**(A)** Overall framework for the testing stage. **(B)** Clinical evaluation of the testing data set and Segmentation model ROC-curve and AUC score analysis. Number of neuroradiologists are 3. ^***^P < 0.001.

The workflow of clinical evaluation (Figure 5) and results of the clinical acceptability analysis of the DLS are presented in Table 5. In this analysis, the majority [85 of 90 (94.0%)] of the DLS-generated segmentations were deemed satisfactory by the experts (no revision required, *n* = 37; minor revision, *n* = 47) (Table 3). Only four patients were assessed to require major revision, with two patients having clinically unacceptable segmentation results. In the assessment of the interrater agreement between the three neuroradiologists for the 90 test patients, the Kendall W test produced a score of 0.006 (*p* = 0.605) indicating a good inter-rater agreement.

**Table 5:** Clinical evaluation 1.

	**Physician 1**	**Physician 2**	**Physician 3**
Perfect (score 3)	34	33	45
Minor revision (score 2)	54	53	35
Major revision (score 1)	1	3	8
Not acceptable (score 0)	1	1	2

## Discussion

In this paper, based on a large dataset of FLAIR images from more than 1,000 patients and with more than 50,000 lesions, we trained a DLS for automatic, and objective segmentation of WMHs. The proposed system was evaluated using pixel-wise and lesion-wise dice scores on internal and external test datasets. The results indicated that the proposed DLS achieved a consistent performance across both the test datasets, indicating good generalizability in the segmentation of WMH from different data sources. Furthermore, in the clinical acceptance analysis, with the 95% acceptance rate by the neuroradiologists, the segmentation results produced by the proposed DLS were observed to have a high clinical acceptance rate. These results collectively indicate that the proposed system can be deployed in clinical practice to quantitatively assess the WMH load in an end-to-end manner with high accuracy and in significantly reduced analysis time. Such a system can aid clinicians in fast and accurate assessment of WMH of the CSVD origin.

### Limitation

This retrospective study analyzed the data from multiple different scanners which could result in a more robust and better generalizable model. However, our analysis did not exhaustively include the data from all the scanners and associated FLAIR image collection protocols, and hence, more extensive testing of the model, in prospective studies is necessary before its adaptation for clinical use. Second, our DLS system for segmentation of WMHs is solely based on MRI-FLAIR imaging features and it does not include complementary information that can be provided by other MRI sequences. Therefore, the possibility of better WMH segmentation using multiple imaging modalities should be explored in future studies.

## Conclusion and Contributions

This study presented a DLS for the segmentation of WMH. Our findings indicate that the DLS can segment the WMHs with good accuracy and significantly smaller analysis time, minimizing the need for the physicians to perform repetitive tasks associated with segmentation. Additionally, the DLS model can reduce intra- and inter neuroradiologists' variation.

## Data Availability Statement

The original contributions presented in the study are included in the article/Supplementary Material, further inquiries can be directed to the corresponding author/s.

## Ethics Statement

The studies involving human participants were reviewed and approved by the Beijing Tiantan Ethics Committee. The patients/participants provided their written informed consent to participate in this study.

## Author Contributions

WS and YD wrote the initial draft of the manuscript, provided both figures and made preliminary revisions. YZ and ZW contributed to DLS development and medical test organization. YD, QW, ZW, PG, YW, and KH made preliminary revisions to the manuscript. YL, KH, and YW made crucial revisions to the manuscript. YZ main contributor for the model development. All authors planned the manuscript, critically revised the initial draft, and made final improvements prior to submission.

## Funding

This study was funded by the China National Neurological Clinical Research Centre, Beijing Postdoctoral Research Foundation (ZZ 2019-09), and China Postdoctoral Science Foundation (No. 2019M660719).

## Conflict of Interest

The authors declare that the research was conducted in the absence of any commercial or financial relationships that could be construed as a potential conflict of interest.

## Publisher's Note

All claims expressed in this article are solely those of the authors and do not necessarily represent those of their affiliated organizations, or those of the publisher, the editors and the reviewers. Any product that may be evaluated in this article, or claim that may be made by its manufacturer, is not guaranteed or endorsed by the publisher.
